# Nosocomial Infection due to Rotavirus in Infants in Alzahra Hospital, Isfahan, Iran

**Published:** 2007-06

**Authors:** Roghayeh Kordidarian, Roya Kelishadi, Yaaghob Arjmandfar

**Affiliations:** Pediatrics Department, Alzahra Hospital, Isfahan University of Medical Sciences, Hezar Jerib St., Isfahan, Iran

**Keywords:** Cross infections, Cross-sectional studies, Diarrhoea, Infantile, Prevalence, Rotavirus, Iran

## Abstract

Rotavirus is one of the most common causes of acute diarrhoea during infancy, and the spread of this infection due to rotavirus in paediatric wards can cause acute diarrhoea during hospitalization, and, in turn, prolong hospitalization or rehospitalization. It is, therefore, important to evaluate the problem and to find an appropriate approach to decrease the rate of infection. The incidence of nosocomial infection due to rotavirus was studied in 80 children aged 3-24 months from November 2003 to April 2004 in the Alzahra Hospital, Isfahan, Iran. Rotavirus antigen was detected by latex aggulutation in stool samples obtained during hospitalization and up to 72 hours after discharge from the hospital. The prevalence of nosocomial infection due to rotavirus was 26.25%, which is a considerable prevalence compared to similar studies which reported a prevalence of 27.7%, 19.4%, and 14.6%. Overall, 15% of the 21 children with positive rotavirus antigen in their stools had acute diarrhoea during hospitalization and up to 72 hours after discharge (symptomatic nosocomial infection), and 11.25% of all children (n=80) studied had asymptomatic nosocomial infection. Regarding the low frequency of nosocomial infection due to rotavirus in other studies which have only studied symptomatic cases during hospitalization and reported a prevalence of 3.3 and 9%, it is suggested that the real estimation of nosocomial infection due to rotavirus in asymptomatic cases that might become symptomatic after discharge from hospital should also be considered. Due to the relatively high frequency of nosocomial infection in the Alzahra Hospital, it is necessary to follow stricter health issues, e.g. isolation of patients with diarrhoea and hand-washing before and after the examination of every patient.

## INTRODUCTION

Diarrhoea is best defined as an excessive loss of fluid and electrolytes in stool (>5 g/kg of stool output per day). It is a common disease of childhood that accounts for a high percentage (10-15%) of causes of hospitalization in paediatric wards and annual mortality of nearly two million children worldwide (1–3).

The major causes of acute diarrhoea are infectious factors, with viruses being the most common. Among viruses, rotavirus is one of the most common causes of diarrhoea, resulting in more than 125 million episodes of acute diarrhoea and 600 thousand deaths per year among children aged less than five years, worldwide. This virus is also considered to be the most frequent aetiological agent of nosocomial infections due to diarrhoea (4–6).

Twenty to 50% of gastroenteritis caused by rotavirus are of nosocomial origin (7–8). This infection is more prevalent and intensive during the first two years of life (1,9). It is common in the colder months (7–10), and oral-faecal contamination is also possible. As the disease provides short-term immunity, recontamination is not uncommon. Spreading of this infection is common in hospitals and childcare centres. It is considered to be the most important factor in hospital-acquired diarrhoea which increases the duration of hospitalization and/or rehospitalization. Nosocomial infections, or hospital-acquired infections, are those acquired during hospitalization and become evident after discharge (11–13).

Regarding the 48-72-hour incubation period of rotavirus, for the assessment of its role on nosocomial infection, it is necessary to examine the child up to 48 hours after discharge from hospital; this is considered as an acquired asymptomatic case. Its importance is due to contamination of hospital staff and the necessity of following simple health points that would result in prevention and control of overflowed infection in hospitals. Considering the importance of this infection and lack of exact information about rotavirus in our community, the study was conducted to evaluate the prevalence of nosocomial infections due to rotavirus to adopt efficient approaches to decrease the rate of contamination (14).

## MATERIALS AND METHODS

This cross-sectional study was conducted during six cold months (November 2003 to April 2004) in the Alzahra Hospital, the main referral hospital in Isfahan province, Iran. The Alzahra Hospital, with more than 1,000 beds, is the second largest and one of the most modern hospitals in the Middle East (www.alzahra.mui.ac.ir).

The study population comprised all patients, aged 3-24 months, who had been hospitalized in the paediatric ward of the Alzahra Hospital because of diseases other than diarrhoea. Subjects were selected by simple sampling from among those children who had been admitted to the paediatric nephrology, endocrinology, neurology, gastroenterology, immunology, cardiology, and haematology wards. Those patients who had negative rotavirus antigen in stool examination on the first day of admission were included in the study. If these children became rotavirus antigen-positive in stool obtained during hospitalization and or up to 72 hours after discharge, they were considered to have a nosocomial infection.

Considering the prevalence of 12.8% and 15.3%, respectively, in our pilot study and in a previous survey in Tehran, Iran (15), the necessary sample size to reach a confidence level of 95% was calculated as 75 subjects.

The same paediatrician visited the patients daily. Nurses recorded information about the patients, including sex, age, type of disease, and length of hospitalization. Considering the immunological factor of breastmilk, we included the type of feeding (breast/bottle feeding) as one of the items in the questionnaire.

Stool samples were examined for those patients who had been affected by diarrhoea during hospitalization and/or up to 72 hours after discharge (except the first 24 hours) and for those children who were not affected by diarrhoea during hospitalization and/or up to 72 hours after their discharge. Patients with diarrhoea from 48 hours after hospitalization to 72 hours after discharge and with positive antigen in their stools were considered as symptomatic cases of nosocomial infection.

The stool samples of patients were kept in closed containers in a refrigerator at 4 °C and were rapidly sent to the hospital laboratory, where the samples were examined for rotavirus antigen using latex agglutination (DSL-kit, Italy). This test has a sensitivity of 85-90% and a specificity of 99-100%. Data were stored in a computer database and were analyzed using the SPSS software (version 11.0) (16). The level of significance was set at p<0.05.

## RESULTS

As presented in the Figure 1, stool samples were collected during the first 24 hours of hospitalization. Eleven subjects with positive rotavirus antigen in stools were excluded from the study, and five patients who developed diarrhoea during the first 48 hours of hospitalization were excluded as well. In addition, 29 patients who could not be followed up after their discharge were also excluded from the study. Consequently, 80 subjects—48 (60%) girls and 32 (40%) boys—were included in the study.

**Fig.**
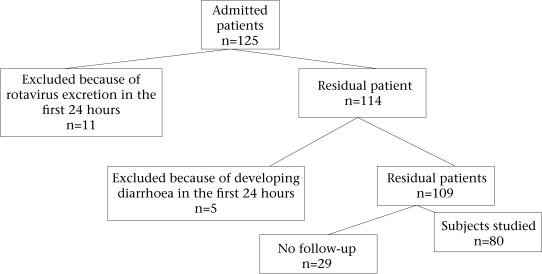
Flow-chart of inclusion-exclusion of subjects in the study

The mean duration of hospitalization was 11 (range 5-25) days. Overall, 21 (26.25%) of the 80 subjects had positive antigen in their stools in the first 72 hours after discharge and were considered as cases of nosocomial infection due to rotavirus. Of them, 13 (16.25%) were affected during hospitalization, and eight (15%) were affected during the first 72 hours after discharge.

Overall, 12 (60%) of the 21 children with nosocomial infection during hospitalization and/or up to 72 hours after discharge developed diarrhoea. The prevalence of symptomatic cases of nosocomial infection was 57.14%, which involved 15% of the study subjects, showing a prevalence of 42.86% for asymptomatic infection that consisted of 11.25% of the total subjects. Eight of the 12 subjects with symptomatic nosocomial infection were affected by diarrhoea during hospitalization and four after discharge.

Fifty-eight (72.5%) subjects were breastfed, and 22 (27.5%) were fed with other types of milk; the two groups had no significant difference in the frequency of nosocomial and/or symptomatic infection due to rotavirus (Table).

**Table:** Comparison of frequency of nosocomial infection due to rotavirus in breastfed and non-breastfed infants

Type of feeding		Nosocomial infection					
	Feeding of infants		Total		Symptomatic case		Asymptomatic case	
	No.	%	No.	%	No.	%	No.	%
Breastfed	58	72.5	15	25.9	8	13.8	7	12.1
Non-breastfed	22	27.5	6	27.3	4	18	2	9.3
Total	80	100	21	6.2	12	15	9	11.2

## DISCUSSION

The study was conducted to assess the relative frequency of nosocomial infection due to rotavirus in hospitalized children aged 3-24 months in the paediatric wards of the main referral hospital of the Isfahan province, Iran. In our study, the prevalence of this infection was 26.25%, of which 15% were symptomatic. In Iran, very few studies have been performed on the prevalence of rotavirus-associated infection in diarrhoeal children. In one study, rotavirus antigen was found in 24.06% of 295 children with acute gastroenteritis and in 2.38% of controls (healthy vector); the type of milk consumed had no effect on this prevalence (17).

In another study at the Pasteur Institute in Tehran, rotavirus was found in 15.3% of 704 children, aged less than five years, with clinical symptoms of acute gastroenteritis and in 1.1% of controls. In this study, the rate of infection was significantly lower in breastfed children than in others, but none of them had been studied for nosocomial infection due to rotavirus (15).

Studies conducted in other countries found a pre-valence of 8-33% of nosocomial infection (4,14). The prevalence of rotavirus-associated nosocomial infection reported in Italy was 27.7% (14), 14.6% in Australia (18), and 19.4% in France (19). The relative frequency of this infection is considerably higher in our study than these studies. The methods of these studies were similar to ours, and the differences in the frequencies in different studies may be due to some epidemiologic issues, such as isolation of patients with diarrhoea and following health rules in different hospitals.

In our study, 15% of the total cases studied had symptomatic nosocomial infection due to rotavirus, whereas in the study in Italy this prevalence was 16.18% (14). In two other studies in France and Spain, the frequency of rotavirus-associated diarrhoea during hospitalization and before discharge was, respectively, 3.3% and 9% (5,19). Our findings are consistent with findings of previous surveys (14,15,17,18) and emphasize the importance of nosocomial infection due to rotavirus and its symptomatic cases after discharge.

In our study, 11.5% of the subjects, i.e. 42.8 % of children affected with rotavirus, were asymptomatic, whereas, in the study performed in Italy, this prevalence was 10.9% (14). This suggests that, while identifying healthy vectors is of great importance, the real prevalence of nosocomial infection has been underestimated in many previous studies, which have only included subjects affected by diarrhoea during hospitalization, but not outpatient cases. The role of breastfeeding in the prevention of rotavirus-associated infection is documented in some studies (6), but such association remains to be confirmed (1,4).

The present study had some limitations. The major limitation was the short duration of study (6 months). If the study could have been conducted for a whole year, it could have shown the seasonal difference. However, since rotavirus is more prevalent during the autumn and winter seasons and since the study was conducted during the colder months, the results reflect a reliable estimate of this infection. The second limitation is that the study was conducted in only one hospital, but considering the fact that this hospital is the main referral hospital in the province with different subspecialty paediatric wards, the study subjects may be considered to be a good representative sample for the study.

The study showed a high frequency of nosocomial infection due to rotavirus and that most study subjects had acute diarrhoea, while others had an asymptomatic infection which is suggested to be due to contamination. These findings emphasize the importance of nosocomial infections in paediatric wards which, in turn, result in considerable costs of hospitalization and treatment. As documented in some previous studies (19,20), we suggested that hygienic rules should be followed more strictly in all the wards admitting children. Isolation is usually considered only for patients with bloody diarrhoea and suspected to have shigellosis. Regarding the high potential of nosocomial infections due to rotavirus, it is suggested that children with acute diarrhoea be isolated and that emphasis be put on some simple health points, such as hand-washing before and after the examination of each patient and cleaning the examination instruments after use in each patient which can be a great help in decreasing the prevalence of nosocomial infection.
